# *In situ* growing of CoO nanoparticles on g-C_3_N_4_ composites with highly improved photocatalytic activity for hydrogen evolution

**DOI:** 10.1098/rsos.190433

**Published:** 2019-07-10

**Authors:** Xuecheng Liu, Qian Zhang, Liwei Liang, Lintao Chen, Yuyou Wang, Xiaoqing Tan, Li Wen, Hongyu Huang

**Affiliations:** 1 Engineering Research Center for Waste Oil Recovery Technology and Equipment, Chongqing Key Laboratory of Catalysis and New Environmental Materials, College of Environment and Resources, Chongqing Technology and Business University, Chongqing 400067, People's Republic of China; 2 Guangdong Provincial Key Laboratory of New and Renewable Energy Research and Development, Guangzhou Institute of Energy Conversion, Chinese Academy of Sciences, Guangzhou 510640, People's Republic of China

**Keywords:** CoO nanoparticle, photocatalytic hydrogen production, g-C_3_N_4_

## Abstract

CoO/g-C_3_N_4_ hybrid catalyst is facilely prepared for application to photocatalytic H_2_ evolution from water splitting by the vacuum rotation–evaporation and *in situ* thermal method. The physical and chemical properties of CoO/g-C_3_N_4_ are determined by a series of characterization methods. The g-C_3_N_4_ with 0.6 wt% Co loading exhibits superior photocatalytic hydrogen evolution activity with an H_2_ evolution amount of 23.25 mmol g^−1^ after 5 h. The obtained 0.6 wt% CoO/g-C_3_N_4_ can split water to generate 0.39 mmol g^−1^ H_2_ without sacrificial agent and noble metal, while the pure g-C_3_N_4_ is inactive under the same reaction conditions. The remarkable enhancement of photocatalytic H_2_ evolution activity of CoO/g-C_3_N_4_ composites is mainly ascribed to the effective separation of electron–hole pairs and charge transfer. The work creates new opportunities for the design of low-cost g-C_3_N_4_-based photocatalysts with high photocatalytic H_2_ evolution activity from overall water splitting.

## 1. Introduction

The development of photocatalytic water splitting into hydrogen by using solar energy has been considered as one of the most promising strategies in photocatalysis to solve the global energy crisis and environmental deterioration [1–3]. Great efforts have been devoted to searching for cheap and efficient photocatalysts. Graphitic carbon nitride (g-C_3_N_4_) with an appropriate band gap (approx. 2.7 eV) has been assumed to be a promising candidate photocatalyst for hydrogen production [4]. However, the photocatalytic hydrogen production activity of g-C_3_N_4_ is limited by low electrical conductivity, a lack of visible light absorption and high charge carries recombination rate [5,6].

The photocatalytic activity of materials can be improved with the enhanced band structures, light adsorbance, charge transport and photogenerated electron–hole pairs separation [7,8]. Several strategies, such as synthesis techniques [9], nanostructure design [10] and electronic structure modulation [11,12], have been conducted to obtain highly efficient g-C_3_N_4_-based photocatalysts. Apart from the above-mentioned methods, a Z-scheme photocatalytic system with two different photocatalysts which sparked interest from the natural photosynthetic systems of plant leaves is one of the most promising approaches to obtain hydrogen evolution from water splitting. The construction of Z-scheme g-C_3_N_4_-based composite has been widely investigated, e.g. NiO/g-C_3_N_4_ [13], Cu_2_O/g-C_3_N_4_ [14], CeO_2_/g-C_3_N_4_ [15]_,_ TiO_2_/g-C_3_N_4_ [12], Bi_2_MoO_6_/g-C_3_N_4_ [16] and Cr_2_O_3_/g-C_3_N_4_ [17], to promote the photoactivity for water splitting. In comparison with pure g-C_3_N_4_, these Z-scheme g-C_3_N_4_-based composites have superior potential for promoting charge transportation, limiting the photogenerated electron–hole pairs recombination, strengthening light absorbance and lowering the redox overpotential [18]. Recently, the construction of heterojunction photocatalysts is mainly focused on how to effectively limit photogenerated electron–hole pairs recombination, and it places less emphasis on the selection of semiconductors. In fact, in order to optimize the fabrication of Z-scheme g-C_3_N_4_-based photocatalysts for overall water splitting, it is important to design a heterojunction photocatalyst with band energy alignments not only trapping an electron to effectively separate the photogenerated charges but also suppressing the back reaction of water formation.

Cobalt monoxide (CoO), as a traditional transition metal monoxide, has gained more attention for its application to photocatalytic water splitting. It is reported that CoO exhibits good photocatalytic water splitting activity with an extraordinary STH of around 5% [1,19]. Wang and co-workers [20] reported photocatalytic decomposition of water for hydrogen evolution on a CoO/SrTiO_3_ catalyst in 2007. Besides, CoO with efficient photo-induced electrons separation can be used as an effective co-catalyst to improve the photocatalytic water splitting activity for hydrogen evolution [21]. But the poor stability of the CoO catalyst is caused by H_2_O_2_ poisoning, hindering its further development [22–24]. It is still a challenge to seek a suitable structure of CoO-based catalyst with high activity and stability.

It is reported that the combination of g-C_3_N_4_ and CoO can result in improved photocatalysts for water splitting [25–27]. The particles could be well dispersed on the carrier by the vacuum rotation–evaporation method [28]. In this work, CoO nanoparticles are growing *in situ* on the g-C_3_N_4_ to prepare well-dispersed CoO/g-C_3_N_4_ composite photocatalyst by the vacuum rotation–evaporation and thermal annealing methods under nitrogen atmosphere. The physical structure, chemical composition, photoelectric properties and photocatalytic H_2_ generation activity of CoO/g-C_3_N_4_ nanocomposite with different Co loading are investigated in detail by a series of characterizations. The enhancement mechanism of photocatalytic overall water splitting for hydrogen evolution of as-synthesized CoO/g-C_3_N_4_ nanocomposite is also discussed.

## 2. Experimental section

### 2.1. Sample preparation

Urea, triethanolamine and cobalt nitrate with analytical grade are purchased from Aladdin Industrial Corporation (Shanghai, China). Firstly, g-C_3_N_4_ is prepared by the thermal polycondensation of urea [29]. Typically, 10 g urea is placed into a covered crucible and heated at 500°C for 3 h using a heating rate of 10°C min^−1^ in a muffle furnace to obtain g-C_3_N_4_. By sonication, 200 mg g-C_3_N_4_ powder is dispersed in 50 ml of deionized water. According to the mass ratio of Co from 0 to 5%, the certain volume of Co(NO_3_)_2_ aqueous solution is dipped into the prepared g-C_3_N_4_ aqueous dispersion and stirred continuously for 20 h to form homogeneous solution with water bath at 70°C for 12 h. After rotavaporation to dryness, the obtained impregnated sample is annealed at 400°C for 2 h in nitrogen atmosphere in the tube furnace and the CoO nanoparticles are grown *in situ* on the g-C_3_N_4_ sheets to obtain CoO/g-C_3_N_4_ composites.

### 2.2. Sample characterization

The phase compositions of the prepared materials are determined by an X-ray diffractometer (XRD) with Cu Kα radiation (modelD/max RA, RigakuCo., Japan). The transmission electron microscope (TEM) images are obtained by using the electron microscope (JEM-6700F, Japan). X-ray photoelectron spectroscopy (XPS) measurements are analysed by Thermo ESCALAB 250, USA, with Al Ka X-rays radiation operated at 150 W. The XPS spectra of the samples were calibrated by using the C1s level at 284.5 eV as an internal standard. Diffuse reflectance spectra of the dry-pressed disc samples are performed by a UV–Visible spectrometer (UV-2700, Shimadzu, Japan). Photoluminescence (PL) is recorded on a fluorescence spectrometer with an Xe lamp as an excitation source with optical filters (FS-2500, Japan). Electrochemical analysis is carried out on a CHI660E workstation. Electrochemistry impedance spectroscopy (EIS) and photoelectric current response measurements are conducted on a conventional three-electrode system with the as-prepared photocatalyst coated onto a 2 cm × 1 cm FTO glass electrode as a working electrode, platinum foil as a counter electrode and Ag/AgCl as a reference electrode, respectively. The frequency range is from 0.01 Hz to 100 kHz in an alternating voltage of 0.02 V under chopped illumination with 40 s light on/off cycles in 0.1 M Na_2_SO_4_ aqueous solution. Incident light was performed by an Xe arc lamp.

### 2.3. Photocatalytic H_2_ generation testing

Photocatalytic hydrogen evolution reactions are measured in a top-irradiation reaction vessel with a 300 W xenon lamp connected to a closed glass gas-circulation system (CEL-SPH2N, AG, CEAULIGHT). Fifty milligrams of photocatalyst are put into an aqueous solution with 45 ml water and 5 ml triethanolamine. Then, 1.5 wt% of Pt nanoparticles are loaded onto the surface of catalysts *in situ* photo-deposition by using H_2_PtCl_6_·6H_2_O as the precursor. For the overall water splitting, 50 mg of photocatalyst is put into an aqueous solution with 50 ml water without sacrificial agent and noble metal Pt. Next, the reaction system is sealed and evacuated for 30 min to remove air prior to the irradiation experiments, and during the photocatalytic reaction, the reaction solution temperature is kept around 10°C by a flow of cooling water. The outlet gases are analysed by gas chromatography (GC 7920, Beijing) with a thermal conductivity detector and nitrogen as the carrier gas to determine the amount of generated hydrogen.

## 3. Result and discussion

**Figure 1.**
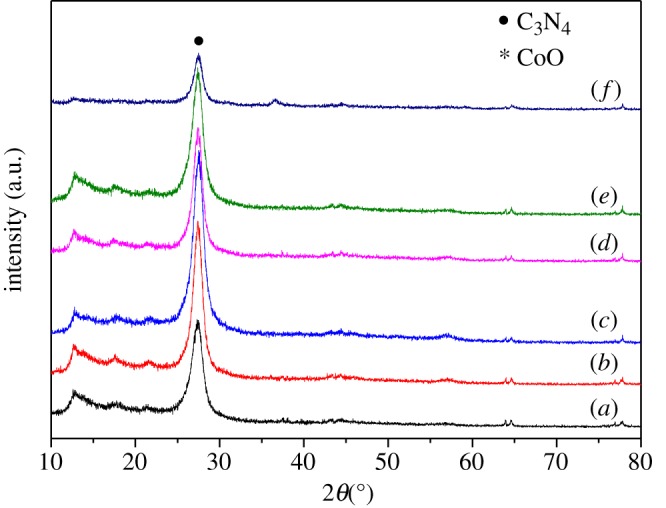
XRD patterns of (*a*) g-C_3_N_4_, (*b*) 0.3% CoO/g-C_3_N_4_, (*c*) 0.6% CoO/g-C_3_N_4_, (*d*) 0.8% CoO/g-C_3_N_4_, (*e*) 1% CoO/g-C_3_N_4_ and (*f*) 5% CoO/g-C_3_N_4_ composites.

The crystalline structures and the phase components of as-prepared CoO/g-C_3_N_4_ composites and g-C_3_N_4_ are studied by XRD. As shown in figure 1, the based materials give two typical diffraction peaks at 13.0° and 27.4°, which can be indexed to the (100) and (002) reflections of g-C_3_N_4_, respectively. It is assumed that g-C_3_N_4_ has a wrinkled sheet-like structure with relatively smooth surface [30]. For CoO/g-C_3_N_4_ composites, the diffraction peaks of g-C_3_N_4_ are observed clearly, indicating that these prepared samples maintain the basic structure of g-C_3_N_4_. But in comparison with pure g-C_3_N_4_, there is a distinct diffraction peak at 36.4°, which can perfectly match with the face-centred cubic CoO structure (JCPDS 71-1178). The characteristic diffraction peaks of both CoO and g-C_3_N_4_ reveal the successful fabrication of CoO/g-C_3_N_4_ composites by *in situ* growing of CoO nanoparticles on g-C_3_N_4_.

**Figure 2.**
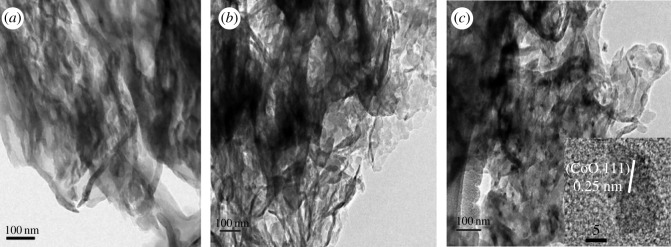
TEM and HR-TEM images of (*a*) g-C_3_N_4_, (*b*) 0.6% CoO/g-C_3_N_4_ and (*c*) 5% CoO/g-C_3_N_4_ composites.

The TEM images of the prepared materials are presented in figure 2. As shown in figure 2*b,c*, the CoO nanoparticles are highly dispersed by *in situ* growing onto the g-C_3_N_4_ matrix. From the enlarged high-resolution TEM of 5% CoO/g-C_3_N_4_ in figure 2*c*, the exposed crystal planes of the obtained CoO can be easily observed, and the lattice fringes with a spacing of 0.25 nm are attributed to the (111) planes of CoO. Based on the XRD and TEM characterizations, this can provide solid evidence for the successful formation of a CoO/g-C_3_N_4_ heterostructure with the *in situ* growing method.

**Figure 3.**
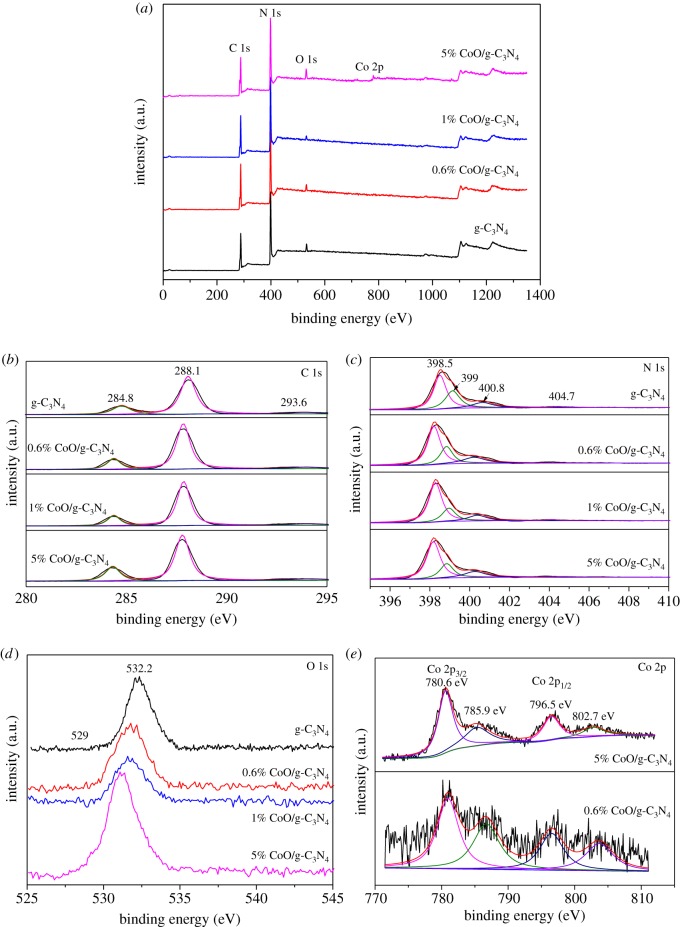
XPS profiles of (*a*) survey, (*b*) C 1s, (*c*) N 1s, (*d*) O 1s and (*e*) Co 2p of the prepared samples.

Surface chemical states of elements and the specific bonding in the prepared samples are characterized in-depth by XPS, and the results are shown in figure 3*a–e*. The full survey spectrum of the prepared material is shown in figure 3*a*. There are three sharp peaks with binding energy values of 285, 399, 530 and 782 eV attributed to the signals of C 1s, N 1s, O 1s and Co 2p, respectively, in the as-prepared samples. The C 1s spectra (figure 3*b*) can be deconvoluted into three peaks at 284.8 eV, 288.1 eV and 293.6 eV, respectively. The binding energy at 284.8 eV can be ascribed to the signals of C–C coordination of the surface adventitious carbon. The binding energy at 288.1 eV is attributed to the *sp*^2^-hybridized carbon in N=C–N coordination, while the peak observed at 293.6 eV results from π-excitation [31]. Figure 3*c* presents the N 1s spectra, which can be subdivided into four peaks at 398.7, 399.4, 400.9 and 404.7 eV. The binding energy at 398.7 eV is ascribed to the *sp*^2^-hybridized nitrogen atoms in C=N–C groups [32]. The binding energy at 399.4 eV is corresponding to the tertiary nitrogen N–C_3_ groups or H–N–C_2_ [33]. The binding energy at 400.9 eV is result from the amino function groups [32], and the binding energy at 404.7 eV is attributed to charging effects or positive charge localization in heterocycles [34]. The high-resolution XPS spectra of Co 2p of 0.6% CoO/g-C_3_N_4_ and 1% CoO/g-C_3_N_4_ are displayed in figure 3*e*. The weak and diffused Co 2p peaks of 0.6% CoO/g-C_3_N_4_ at two pairs of individual peaks centred at 780.3 and 796.2 eV confirm the existence of Co, which are identified as the major binding energies of Co^2+^ in CoO [35]. Two peaks at 780.6 and 796.5 eV can be attributed to the Co 2p_3/2_ and Co 2p_1/2_ spin-orbit peaks of CoO, respectively [1]. The O 1s spectra with two peaks at about 529 and 532 eV are shown in figure 3*d*. The binding energy at 529 eV is ascribed to the Co–O bond in the CoO phase [36], while the strong peak at about 532 eV corresponds to the Co–O–C bond, indicating that a strong interaction exists between CoO and g-C_3_N_4_ [26]. It can be seen that the signal of Co–O–C bond becomes bigger with the increase in Co loading because of the change of electronic state of adsorbed oxygen species [37]. Therefore, the interfacial interaction between CoO and g-C_3_N_4_ could be greatly enhanced due to the interfacial hybridized Co–O–C bond.

**Figure 4.**
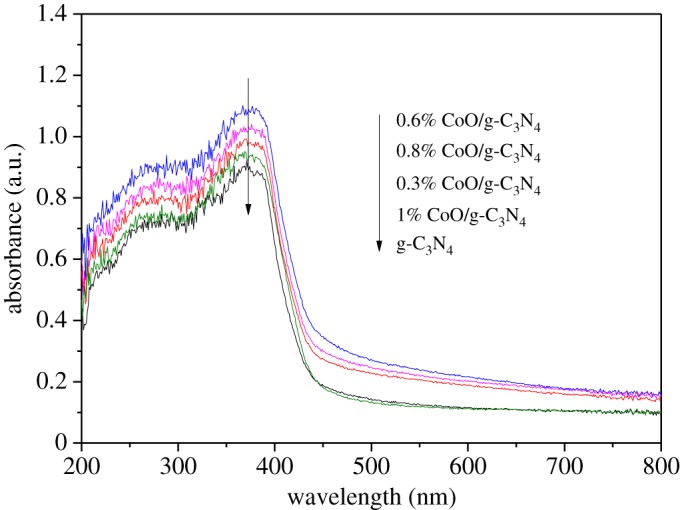
UV–Vis absorbance spectra of g-C_3_N_4_, 0.3% CoO/g-C_3_N_4_, 0.6% CoO/g-C_3_N_4_, 0.8% CoO/g-C_3_N_4_ and 1% CoO/g-C_3_N_4_ composites.

**Figure 5.**
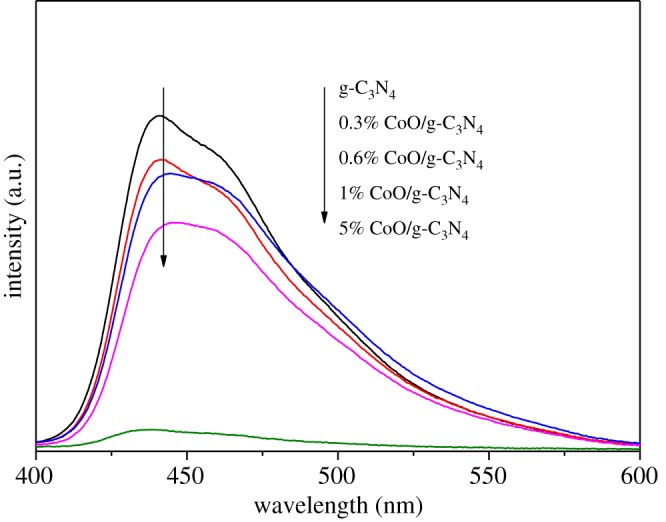
PL spectra (*λ*_ex_ = 370 nm) for the prepared g-C_3_N_4_, 0.3% CoO/g-C_3_N_4_, 0.6% CoO/g-C_3_N_4_, 1% CoO/g-C_3_N_4_ and 5% CoO/g-C_3_N_4_ composite.

It is widely accepted that the optical and photoelectric properties are of great significance to the photocatalytic activity. UV–Vis absorption spectra and PL are measured to identify these properties of g-C_3_N_4_ and CoO/g-C_3_N_4_ composite samples. Figure 4 displays the UV–Vis diffuse reflectance spectra of pure g-C_3_N_4_ and CoO/g-C_3_N_4_ with different CoO contents. It is seen that 0.6% CoO/g-C_3_N_4_ exhibits the best ultraviolet and visible light absorbance, indicating that the 0.6% CoO/g-C_3_N_4_ composite could obtain the best photocatalytic activity for hydrogen evolution by using more solar light. The efficient separation of photo-induced electron–hole pairs is another factor to influence the photocatalytic activity. It is well known that photocatalytic activity is enhanced by interfaces of heterojunctions with a faster separation efficiency of photogenerated electron–hole pairs. The PL analysis is usually carried out to investigate the transfer, migration and recombination of photogenerated electron–hole pairs of the photocatalyst [26]. The PL spectra of as-prepared g-C_3_N_4_ and CoO/g-C_3_N_4_ composites with excitation wavelength of 370 nm are demonstrated in figure 5. CoO/g-C_3_N_4_ composite exhibits a much weaker emission profile with the CoO loading content increasing in comparison with g-C_3_N_4_, indicating that the recombination rate of the photogenerated charge carrier is greatly restrained and there is a faster photoelectron transfer between the hybrid of CoO and g-C_3_N_4_.

**Figure 6.**
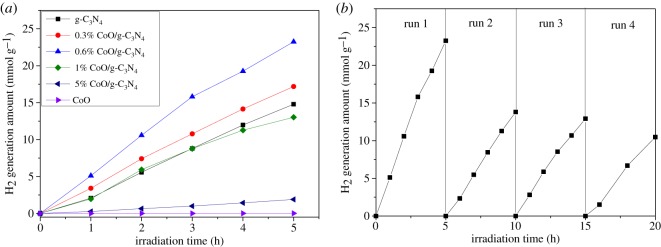
(*a*) The photocatalytic H_2_ evolution amount of the samples; (*b*) recyclability of 0.6 wt% CoO/g-C_3_N_4_ photocatalyst for the photocatalytic H_2_ evolution, with 10 vol% TEOA, 1.5 wt% Pt.

The photocatalytic H_2_ evolution performance of the prepared CoO/g-C_3_N_4_ composite with different CoO content is measured using Pt as a co-catalyst and the results are shown in figure 6. In figure 6*a*, it can be found that the photocatalytic H_2_ evolution amount for CoO/g-C_3_N_4_ composite with 0, 0.3, 0.6, 1, 5 and 100 wt% Co loading content is recorded to be 14.79, 17.19, 23.25, 13.02, 1.90 and 0.019 mmol g^−1^ after 5 h, respectively. The photocatalytic H_2_ evolution amount increases as the Co content increases from 0 to 0.6 wt% and then exhibits a decrease with a higher Co content. This decrease is possible due to excessive CoO aggregation and the decrease in g-C_3_N_4_ surface active sites. The 0.6 wt% CoO/g-C_3_N_4_ composite exhibits the best photocatalytic performance with an average hydrogen evolution rate of 4.65 mmol h^−1^ g^−1^, which is about 57% higher than that of pure g-C_3_N_4_. Compared with the reported 0.5 wt% CoO/g-C_3_N_4_ (0.65 mmol h^−1^ g^−1^) [35], 30 wt% CoO/g-C_3_N_4_ (2.51 µmol h^−1^) [26] and 10 wt% CoO/g-C_3_N_4_ (0.46 µmol h^−1^) [25], photocatalytic hydrogen evolution performance of CoO/g-C_3_N_4_ composite could be improved by the rotation–evaporation and thermal annealing methods. Figure 6*b* shows the photocatalytic stability of hydrogen evolution for the 0.6 wt% CoO/g-C_3_N_4_ sample is carried out by four cycling experiments under the same condition. The photocatalytic H_2_ evolution activity of 0.6% CoO/g-C_3_N_4_ exhibits favourable stability for the four recycling runs.

**Figure 7.**
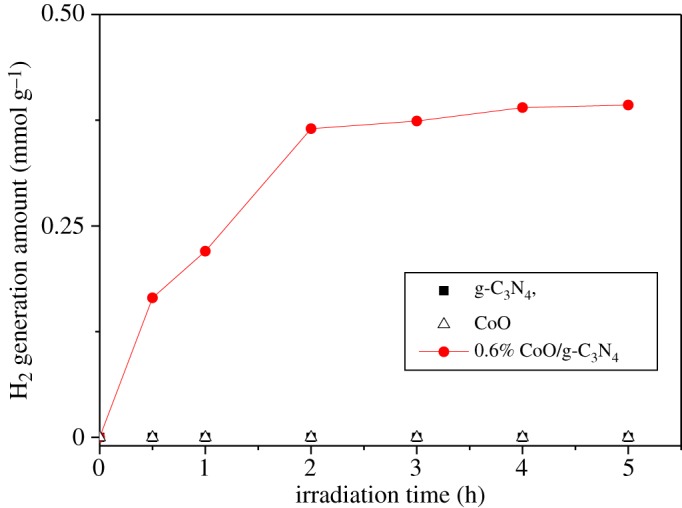
H_2_ evolutions from pure water with g-C_3_N_4_, CoO and 0.6 wt% CoO/g-C_3_N_4_.

In the following experiments, as 0.6 wt% CoO/g-C_3_N_4_ exhibits the superior photocatalytic H_2_ evolution activity, its H_2_ evolution performance for splitting pure water is investigated and the results are shown in figure 7. It is found that 0.6 wt% CoO/g-C_3_N_4_ can split pure water to generate H_2_ without sacrificial agent and noble metal Pt, while the pure g-C_3_N_4_ and bulk CoO exhibit negligible photocatalytic activity towards H_2_ generation under the same reaction condition. H_2_O_2_ is more easily generated than O_2_, which is attributed to the more positive H_2_O_2_/H_2_O (1.78 V versus RHE) than O_2_/H_2_O (1.23 V versus RHE) [26]. The drawbacks of rapid rate of photogenerated electron–hole pairs and severe poisoning by H_2_O_2_ generated during the reaction of g-C_3_N_4_ are the main reasons for the inactivation [13]. The photocatalytic H_2_ evolution amount of 0.6% CoO/g-C_3_N_4_ reaches 0.39 mmol g^−1^ and the photocatalytic H_2_ evolution rate is very slow after 2 h under light irradiation. Based on these results, it can be safely concluded that g-C_3_N_4_ doped with 0.6 wt% CoO could effectively separate the photogenerated electron–hole pairs to generate H_2_ from pure water splitting; however, it is likely subject to being greatly poisoned by H_2_O_2_ generation during the photocatalytic reaction to cause rapid inactivation.

**Figure 8.**
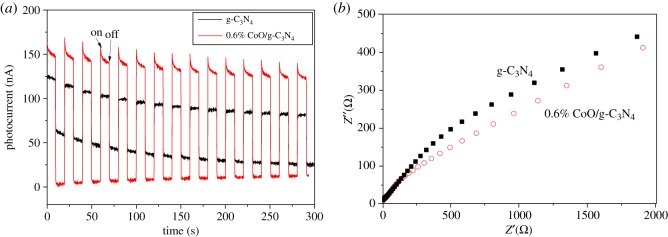
(*a*) Transient photocurrents, (*b*) electrochemical impedance spectra of g-C_3_N_4_ and 0.6% CoO/g-C_3_N_4_ electrodes at 0.3 and −0.4 V versus Ag/AgCl.

The photogenerated charge transfer and separation properties in the 0.6 wt% CoO/g-C_3_N_4_ sample are further studied by photoelectrochemical measurements. EIS and photocurrents are carried out to obtain the photogenerated charge separation and transfer properties. Figure 8*a* shows the transient photocurrent responses for g-C_3_N_4_ and 0.6% CoO/g-C_3_N_4_ samples with an interval light on/off cycle mode. It can be clearly observed that the transient photocurrent response of 0.6% CoO/g-C_3_N_4_ composite is higher than that of the pure g-C_3_N_4_ sample, suggesting that the interfical electron transfer and electron–hole separation process between CoO and g-C_3_N_4_ is highly proven. The EIS measurements have been carried out to evaluate the photogenerated electron transfer in CoO/g-C_3_N_4_. Figure 8*b* presents the experimental Nyquist impedance plots for g-C_3_N_4_ and 0.6% CoO/g-C_3_N_4_ samples in the dark. The Nyquist plot of 0.6% CoO/g-C_3_N_4_ suggests an apparently smaller arc diameter than that of g-C_3_N_4_, indicating that the 0.6% CoO/g-C_3_N_4_ system can efficiently migrate interfacial charge and separate the photogenerated electron–hole pairs at the heterojunction interface across the electrode/electrolyte in agreement with the results of PL, contributing to the enhancement of photocatalytic hydrogen evolution activity [26].

**Figure 9.**
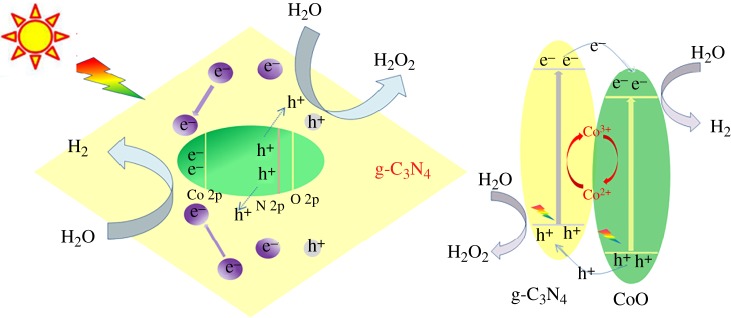
Schematic illustrations of the proposed photocatalytic H_2_ evolution mechanism for overall water splitting over the CoO/g-C_3_N_4_ hybrid catalyst.

Based on the above experimental results, a possible mechanism of photocatalytic H_2_ evolution for the CoO/g-C_3_N_4_ hybrid system is proposed, as shown in figure 9. First, the electron–hole pairs [38,39] are generated in the conduction band [40] and valence band of g-C_3_N_4_ by light irradiation. Then, the photogenerated holes in the valence band of g-C_3_N_4_ will react with H_2_O to generate H_2_O_2_. In contrary, the photogenerated electrons further transfer from the conduction band of g-C_3_N_4_ to the surface of CoO nanoparticles, which function as reduction catalysts to catalyse the reduction in protons (H^+^) to H_2_. Therefore, the separation of electron–hole pairs and the charge transfer can effectively enhance the photocatalytic H_2_ evolution activity from overall water splitting for the CoO/g-C_3_N_4_ heterojunction photocatalyst.

## 4. Conclusion

The CoO/g-C_3_N_4_ hybrid catalysts with different CoO loading contents are facilely prepared to study the photocatalytic H_2_ evolution activity. CoO/g-C_3_N_4_ composite with 0.6 wt% Co loading shows the highest photocatalytic activity for H_2_ evolution amount of 23.25 mmol g^−1^ after 5 h, which is 57% higher than that of g-C_3_N_4_. The remarkably enhanced photocatalytic performance for H_2_ evolution of CoO/g-C_3_N_4_ composite is mainly due to the faster transfer of interfacial charge and more effective separation of electron–hole pairs. The photocatalytic H_2_ evolution amount of 0.6% CoO/g-C_3_N_4_ reaches 0.39 mmol g^−1^ by overall water splitting without sacrificial agent and noble metal. But the photocatalytic H_2_ evolution rate of 0.6% CoO/g-C_3_N_4_ is very slow after 2 h because it is easily poisoned by H_2_O_2_ generation during the photocatalytic reaction to cause rapid inactivation. In future work, CoO/g-C_3_N_4_ material with the stability of photocatalytic H_2_ evolution activity will be further designed to prevent H_2_O_2_ poisoning.

## Supplementary Material

Reviewer comments

## Data accessibility

Data available from the Dryad Digital Repository at: https://doi.org/10.5061/dryad.cs0sj00 [31].
